# Automated computed tomographic scoring of lung disease in adults with primary ciliary dyskinesia

**DOI:** 10.1186/s12890-018-0758-6

**Published:** 2018-12-18

**Authors:** Trieu-Nghi Hoang-Thi, Marie-Pierre Revel, Pierre-Régis Burgel, Laurence Bassinet, Isabelle Honoré, Thong Hua-Huy, Charlotte Martin, Bernard Maitre, Guillaume Chassagnon

**Affiliations:** 1 Radiology Department, Groupe Hospitalier Cochin-Hôtel Dieu, AP-HP, Université Paris Descartes - Sorbonne Paris Cité, Paris, France; 2 Pulmonary Department, Groupe Hospitalier Cochin-Hôtel Dieu, AP-HP, Université Paris Descartes - Sorbonne Paris Cité, Paris, France; 3 Service de Pneumologie et de Pathologie Professionnelle, DHU A-TVB, Centre Hospitalier Intercommunal de Créteil, Université Paris Est Créteil, Créteil, France; 4 Physiology Department, Groupe Hospitalier Cochin-Hôtel Dieu, AP-HP, Université Paris Descartes - Sorbonne Paris Cité, Paris, France; 5 Center for Visual Computing, CentraleSupelec, Gif-sur-Yvette, France; 6 Department Diagnostic Imaging, Vinmec International Hospital – Central Park, Ho Chi Minh City, Vietnam

**Keywords:** Computed tomography, Primary ciliary dyskinesia, Kartagener syndrome, Bronchiectasis

## Abstract

**Background:**

The present study aimed to develop an automated computed tomography (CT) score based on the CT quantification of high-attenuating lung structures, in order to provide a quantitative assessment of lung structural abnormalities in patients with Primary Ciliary Dyskinesia (PCD).

**Methods:**

Adult (≥18 years) PCD patients who underwent both chest CT and spirometry within a 6-month period were retrospectively included. Commercially available lung segmentation software was used to isolate the lungs from the mediastinum and chest wall and obtain histograms of lung density. CT-density scores were calculated using fixed and adapted thresholds based on various combinations of histogram characteristics, such as mean lung density (MLD), skewness, and standard deviation (SD). Additionally, visual scoring using the Bhalla score was performed by 2 independent radiologists. Correlations between CT scores, forced expiratory volume in 1 s (FEV_1_) and forced vital capacity (FVC) were evaluated.

**Results:**

Sixty-two adult patients with PCD were included. Of all histogram characteristics, those showing good positive or negative correlations to both FEV_1_ and FVC were SD (*R* = − 0.63 and − 0.67; *p* < 0.001) and Skewness (*R* = 0.67 and 0.67; *p* < 0.001). Among all evaluated thresholds, the CT-density score based on MLD + 1SD provided the best negative correlation with both FEV_1_ (*R* = − 0.68; *p* < 0.001) and FVC (*R* = − 0.71; *p* < 0.001), close to the correlations of the visual score (*R* = − 0.60; *p* < 0.001 for FEV_1_ and *R* = − 0.62; *p* < 0.001, for FVC).

**Conclusions:**

Automated CT scoring of lung structural abnormalities lung in primary ciliary dyskinesia is feasible and may prove useful for evaluation of disease severity in the clinic and in clinical trials.

## Background

Primary ciliary dyskinesia (PCD) is a rare genetic disorder characterized by defective ciliary structure and/or function, leading to inadequate mucociliary clearance and chronic oto-sino-pulmonary disease [1–3]. Organ laterality is also affected in almost half the patients [2]. Defective mucociliary airway clearance leads to recurrent and chronic bacterial infections of the lower respiratory tract, and to bronchiectasis [2].

Computed tomography (CT) is the gold standard method for the diagnosis of bronchiectasis, but its utility for monitoring PCD is not yet established [4, 5]. Correlations between CT structural changes and disease severity (lung function) have rarely been studied in PCD, especially in adults [5–13]. However, a large retrospective study recently suggested that a larger disease burden on CT may predict lung function decline in adults with PCD, indicating that CT assessment of lung structural abnormalities might be of value [5].

Most authors who have attempted to quantify bronchial disease in patients with PCD have used visual scoring methods initially designed to assess lung structural changes in patients with cystic fibrosis (CF), but the correlation between these visual scores and forced expiratory volume in 1 s (FEV_1_) remains controversial in patients with PCD [7–14]. For example, Boon et al. reported a good negative correlation between a visual CT score and FEV_1_ (*R* = − 0.63, *P* < 0.001), whereas Cohen-Cymberknoh et al.found no correlation at all (*R* = − 0.36, *P* = 0.61) [11, 12].

Although bronchiectasis, bronchial wall thickening, mucus plugging and mosaic perfusion are present in both PCD and CF, their relative predominance differs between the two diseases. Mosaic perfusion and small-airway mucus plugging predominate in PCD, meaning that their respective weight in the overall CT score should not be the same as in CF [5, 11]. This may be why some authors failed to find a correlation between visual scores and spirometry in PCD patients. Furthermore, visual scores suffer from several limitations, including the need for dedicated training and subjectivity in the assessment of CT changes [15].

Most lung structural changes in PCD, especially bronchial wall thickening, mucus plugging, consolidations and atelectasis are likely to increase lung attenuation and to modify the density histogram characteristics, which can be extracted from the CT images. On the density histogram, the mode corresponds to the most highly represented attenuation value; and skewness describes the asymmetry of the density curve, which is shifted to the right when there is an increase of lung density or to the left in case of decrease. Additionally, high-attenuating structures can be quantified by using a thresholding approach similar to that used to measure emphysema on CT, except that the latter is based on the quantification of low-attenuating lung areas, with attenuation values below minus 950 Hounsfield units (HU) [16]. The quantification of high-attenuating structural changes in the lungs, also using a thresholding approach has been reported to show good correlation with FEV_1_ in patients with CF [17].

We postulated that disease severity in PCD might also be assessed by quantifying high-attenuating lung structures and by analysing changes in the lung density distribution. We therefore developed an automated CT scoring method based on histogram characteristic analysis and threshold-based quantification of high-attenuating lung structures in patients with PCD.

## Methods

### Patients

This retrospective study, performed in two accredited PCD reference centres, was approved by the Institutional Review Board of Société Pneumologie de Langue Française. The need for informed consent was waived, in accordance with French rules for retrospective observational studies.

All adult outpatients, with a diagnosis of PCD according to the ERS guidelines [18] were eligible if they had both chest CT exams of the whole thorax performed between November 2009 and July 2016 and spirometric measurements, both performed within a 6-month period. Exclusion criteria were the unavailability of CT images with a slice thickness ≤ 2 mm, reconstructed with a soft kernel, or the administration of iodinated contrast medium during the CT acquisition.

### CT examinations

All CT examinations had been performed in the supine position at full inspiration, with usual acquisition parameters, allowing obtaining high resolution CT images of the whole thorax during a single breath hold. Five different 16-to-64 multislice CT devices from two different vendors (Somatom Sensation 16 and Somatom Definition DS, Siemens Healthcare, Erlangen, Germany; Lightspeed plus, Bright Speed 16 and Optima CT 660, GE Healthcare, Milwaukee, Wi) had been used, depending on the site and date of the CT examinations, all performed with equivalent acquisition parameters. The radiation dose resulting from each CT acquisition was evaluated by collecting the mean dose-length product (DLP) value from the dose reports.

### Image analysis

Pulmonary situs type was identified as *solitus*, *inversus* or heterotaxy, based on the relationship between the upper-lobe bronchus and the ipsilateral pulmonary artery, and the morphology of the tracheobronchial tree [19]. CT images were also checked for prior lobectomy.

Lung structural changes were assessed by visual scoring and also by histogram analysis and thresholding of high attenuating lung structures.

#### Visual CT scoring was performed as follow

All the images were scored by one thoracic radiologist (CM) using the Bhalla score [20]. Twenty randomly selected examinations were also independently scored by a second radiologist (GC) to assess interobserver repeatability.

#### Automated CT scoring was performed as follows

First, the lungs were isolated from the mediastinum and chest wall using a commercially available, automated lung segmentation software (Myrian XP lung software version 1.19.1,Intrasense, Montpellier, France).

This allowed obtaining isolated whole lung volumes, for further density histogram analysis.

We also obtained separate volumes of the upper (right upper lobe and upper part of the left upper lung) and lower lungs (middle lobe, lingula, and lower lobes), after manual contouring of the fissures. This was only done for further comparison of the upper and lower lung CT-density scores. Otherwise, the process was fully automated.

The following histogram characteristics were analysed: mean lung density (MLD), mode (the most highly represented attenuation value), standard deviation (SD), kurtosis (sharpness of the density distribution), and skewness (asymmetry of the density distribution).

Lung structural changes having high attenuation values were quantified with a thresholding method, in order to obtain a CT-density score. Several threshold values were tested for their correlation with FEV_1_ and forced vital capacity (FVC). Three fixed threshold values were tested (− 300, − 400 and − 500 HU), as well as eight adapted threshold values taking into account, for each CT examination, individual histogram features, known to be influenced by the inspiratory level [21, 22]. We hypothesized that adapted thresholds based on Mode or MLD or integrating SD might compensate for the changes of density distribution related to the level of inspiration.

The CT-density scores (one for each tested threshold value) were expressed as the proportion of lung showing attenuation values above the selected threshold. For instance, a CT-Density score value of 10 indicated that 10% of the lung had an attenuation value superior or equal to the threshold on CT.

More details about the whole procedure can be found on a previous work dedicated to automated scoring of CF lung structural changes [17].

### Pulmonary function tests

Forced vital capacity (FVC) and forced expiratory volume in 1 s (FEV_1_), expressed as the percentage of predicted values, were retrieved from the patients’ files. Spirometry was performed as recommended by the American Thoracic Society/European Respiratory Society [23] and predicted values were calculated using the European Community for Steel and Coal reference values [24].

### Statistical analysis

All analyses were done using the ‘R’ statistical software package (version 3.2.4, R Foundation, Vienna, Austria). Spearman’s correlation coefficient was used to evaluate the correlations between visual scores, histogram characteristics, CT-density scores and spirometry measurements (FEV_1_ and FVC). Spearman R values were interpreted as follows: < 0.4 = absent to weak correlation, 0.40–0.59 = moderate correlation, 0.60–0.79 = good correlation, > 0.8 = strong correlation. To evaluate the distribution of high attenuating lung structural changes, the CT-density scores of the upper lung portions (right upper lobe and upper component of the left upper lobe) were compared to those of the lower lung portions (middle lobe, lingula, and lower lobes), using Wilcoxon’s paired test. Intraclass correlation coefficients (ICC) and Bland-Altman plots were used to assess interobserver repeatability of the visual scores. Excellent repeatability was assumed when the ICC was 0.8 or more.

## Results

### Patients

**Fig. 1:**
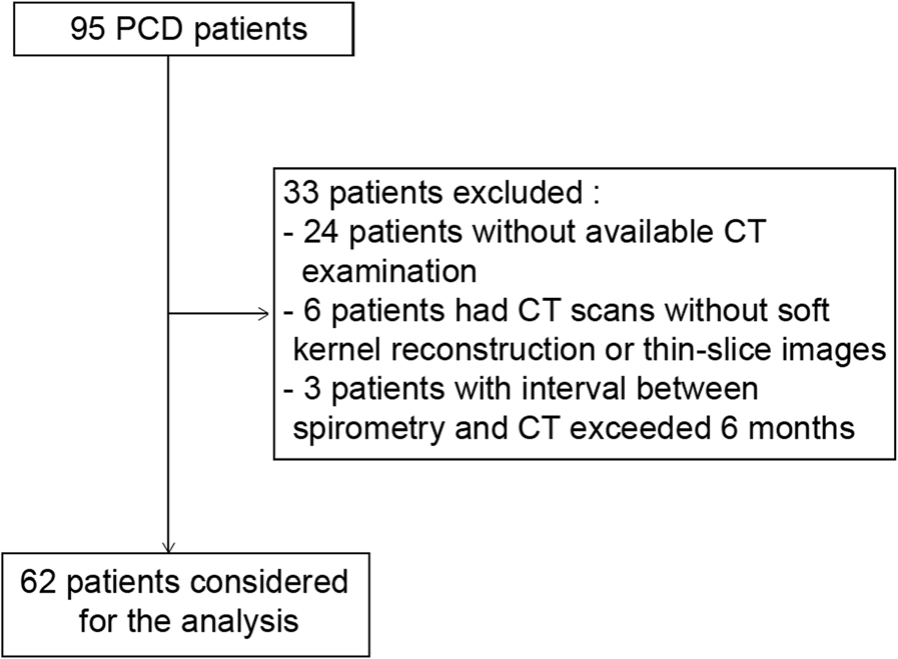
Flow Chart

Between November 2009 and July 2016, 95 patients with a confirmed diagnosis of PCD were identified, of whom sixty-two patients were included in this study. Among the 33 excluded patients, 24 had no available CT examination, 6 patients had CT scans without soft kernel reconstruction or thin-slice images, and the interval between spirometry and CT exceeded 6 months in the remaining 3 cases (Fig. 1). For the 62 patients who were finally included, PCD diagnosis had been confirmed in 51 by electron microscopy of ciliary ultrastructure. The remaining 11 patients had Kartagener’s syndrome with diffuse bronchiectasis and situs inversus on CT imaging, a combination of signs considered to validate PCD diagnosis [13].

Characteristics of the study population are presented in Table 1.

**Table 1:** Characteristics of the patients (*n* = 62)

Male/Female	32 / 30
Age, yr	39 (15)
Bronchial situs	
- solitus	37 (60)
- inversus	24 (39)
- left isomerism	1 (1)
Body mass index, kg/m^2^	23.71 (4)
Percentage of predicted FEV_1_	67 (20)
Percentage of predicted FVC	80 (18)
Median interval between spirometry and CT, months [interquartile range]	0 [0–16]

For quantitative variables, data are mean with standard deviation in parentheses

For qualitative variables, data are numbers of patients, and numbers in parentheses are percentages

*Definition of abbreviations: CT* computed tomography, *FEV*_*1*_ forced expiratory volume in 1 s, *FVC* forced vital capacity

The mean ± SD age was 39 ± 15 years. Mean predicted FEV_1_ was 67 ± 20% and mean predicted FVC was 80 ± 18%. Their correlation to patients’ age was weak (*R* = − 0.32, *p* = 0.012 for FEV_1_) and (*R* = − 0.33, *p* = 0.008 for FVC).

Regarding other characteristics, 37 patients (60%) had the usual arrangement of the pulmonary situs (*situs solitus*), while 24 (39%) had *situs inversus* and 1 (1%) had left isomerism.

Nineteen patients (31%) had previously undergone complete or partial lobectomy. The resections concerned the middle lobe in 15 patients (24%), the left lower lobe in 1 patient (2%), the middle lobe plus the left lower lobe in 2 patients (3%), and the middle lobe plus the lingula in the remaining patient (2%).

### CT examinations

The median interval between CT and spirometry was 0 days [interquartile range: 0–29], 41 of the 62 CT scans being performed on the same day as spirometry. The mean DLP per CT scan was 200.3 ± 100.4 mGy.cm.

### Visual CT score

The interobserver repeatability for the visual score was excellent (ICC = 0.84). Visual CT score, performed for all CT scans by one of the 2 radiologists, showed good correlation with FEV_1_ (*R* = − 0.60; *p* < 0.001) and FVC (*R* = − 0.62; *p* < 0.001).

### Histogram characteristics

**Fig. 2:**
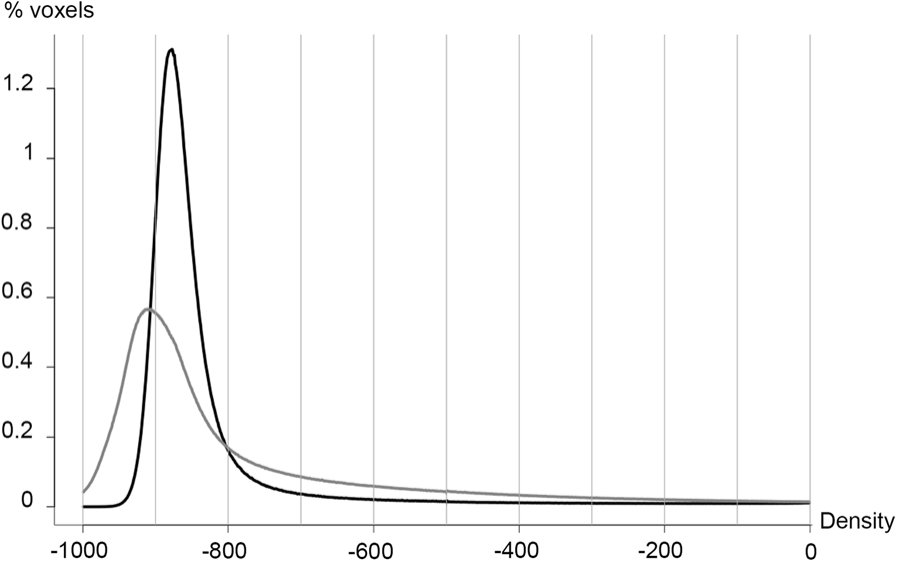
Variation of histogram characteristics according to lung disease severity. Histogram of lung densities in a patient with mild disease (black line; FEV_1_ = 81%; FVC = 101%;SD = 110.1; kurtosis =31.4). The histogram of lung densities in a patient with severe disease (grey line; FEV_1_ = 25%; FVC = 54%) demonstrates higher scattering (SD = 213.9) and flattening (kurtosis = 6.7) of the curve

Two histogram characteristics – kurtosis (*R* = 0.56; *p* < 0.001) and skewness (*R* = 0.60; *p* < 0.001)– showed a moderate to good correlation to FEV_1_ (Table 2). The same two characteristics also correlated with FVC (*R* = 0.65; *p* < 0.001 and 0.67; *p* < 0.001, respectively). SD showed good negative correlation to both FEV1 (*R* = − 0.63; *p* < 0.001) and FVC (*R* = − 0.67; *p* < 0.001). Overall the correlations with FVC were slightly stronger than the correlations with FEV_1_. Examples of variations in histogram shape according to pulmonary function are shown in Fig. 2.

**Table 2:** Correlations between spirometry, histogram characteristics and CT-density scores

	FEV_1_		FVC	
	R	*P* value	R	*P* value
Histogram characteristics				
- MLD	−0.22	0.084	− 0.31	0.012
- Mode	0.14	0.285	0.11	0.397
- SD	−0.63	< 0.001	− 0.67	< 0.001
- Kurtosis	0.56	< 0.001	0.65	< 0.001
- Skewness	0.60	< 0.001	0.67	< 0.001
CT-density score with fixed thresholds				
- (−) 400 HU	−0.51	< 0.001	−0.59	< 0.001
- (−) 500 HU	−0.53	< 0.001	− 0.61	< 0.001
- (−) 600 HU	− 0.54	< 0.001	−0.61	< 0.001
CT-density score with adapted thresholds				
- MLD + 2 SD	−0.62	< 0.001	−0.67	< 0.001
- MLD + 1 SD	−0.68	< 0.001	− 0.71	< 0.001
- Mode + 500 HU	− 0.57	< 0.001	−0.64	< 0.001
- Mode + 400 HU	−0.60	< 0.001	− 0.65	< 0.001
- Mode + 300 HU	− 0.64	< 0.001	−0.70	< 0.001
- Mode + 3 SD	−0.54	< 0.001	− 0.62	< 0.001
- Mode + 2 SD	− 0.65	< 0.001	−0.70	< 0.001
- Mode + 1SD	−0.66	< 0.001	− 0.68	< 0.001

*Definition of abbreviations: CT* computed tomography, *FEV*_*1*_ forced expiratory volume in 1 s, *FVC* forced vital capacity, *HU* Hounsfield unit, *MLD* Mean Lung Density, *SD* Standard Deviation

### CT-density scores

**Fig. 3:**
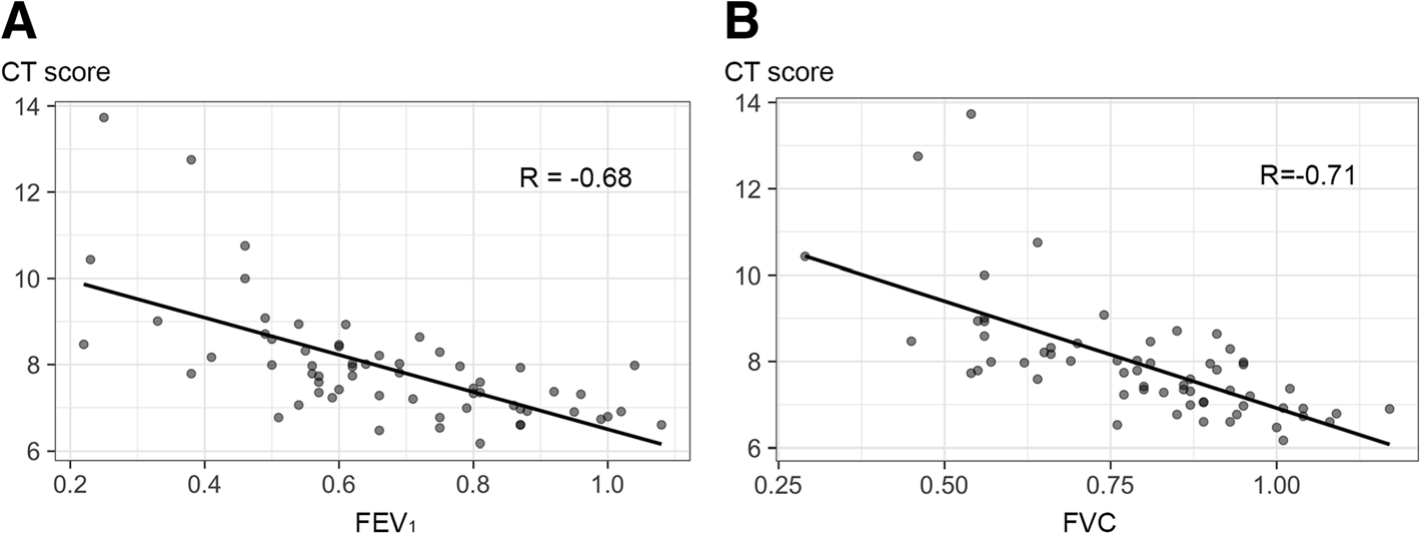
Relationship between the CT-density score based on MLD + 1SD and lung functional parameters. **a** Relationship between CT-density score and FEV_1_. **b** Relationship between CT-density score and FVC

All CT-density scores showed moderate to good negative correlations with FEV_1_ (*R* = − 0.54 to − 0.68; *p* < 0.001) and FVC (*R* = − 0.62 to − 0.71; *p* < 0.001) (Table 2). Overall, CT scores based on fixed thresholds showed weaker negative correlations with FEV_1_ (*R* = − 0.51 to − 0.54; *p* < 0.001) and FVC (*R* = − 0.59 to − 0.62; *p* < 0.001) than did CT-density scores based on adapted thresholds taking into account histogram characteristics. The strongest correlations were obtained using MLD + 1SD as threshold (*R* = − 0.68; *p* < 0.001 for FEV_1_ and *R* = − 0.71 for FVC; *p* < 0.001) (Fig. 3). The correlations between CT score and PFTs were in the same range when considering each center separately: *R* = − 0.66; *p* < 0.001 and − 0.68; *p* < 0.001 for FEV1 and R = − 0.68; *p* < 0.001 and − 0.70; *p* < 0.001 for FVC. With this threshold value (MLD + 1SD), the automated score (CT-density score) correlated well with the visual score (*R* = 0.70, *p* < 0.001). Within the Bhalla visual score, the automated CT-density score correlated well with air wall thickening and mucus plugging- related items (*R* =  0.64 and  0.61, respectively; *p* < 0.001) and moderately with bronchiectasis-related items (*R* =  0.58; *p* < 0.001)”.

The automated score values were significantly higher in the lower lungs (median: 8.83; interquartile range: 7.61–10.06) than in the upper lungs (median: 6.25; interquartile range: 5.57–6.81) (*p* < 0.001).

**Fig. 4:**
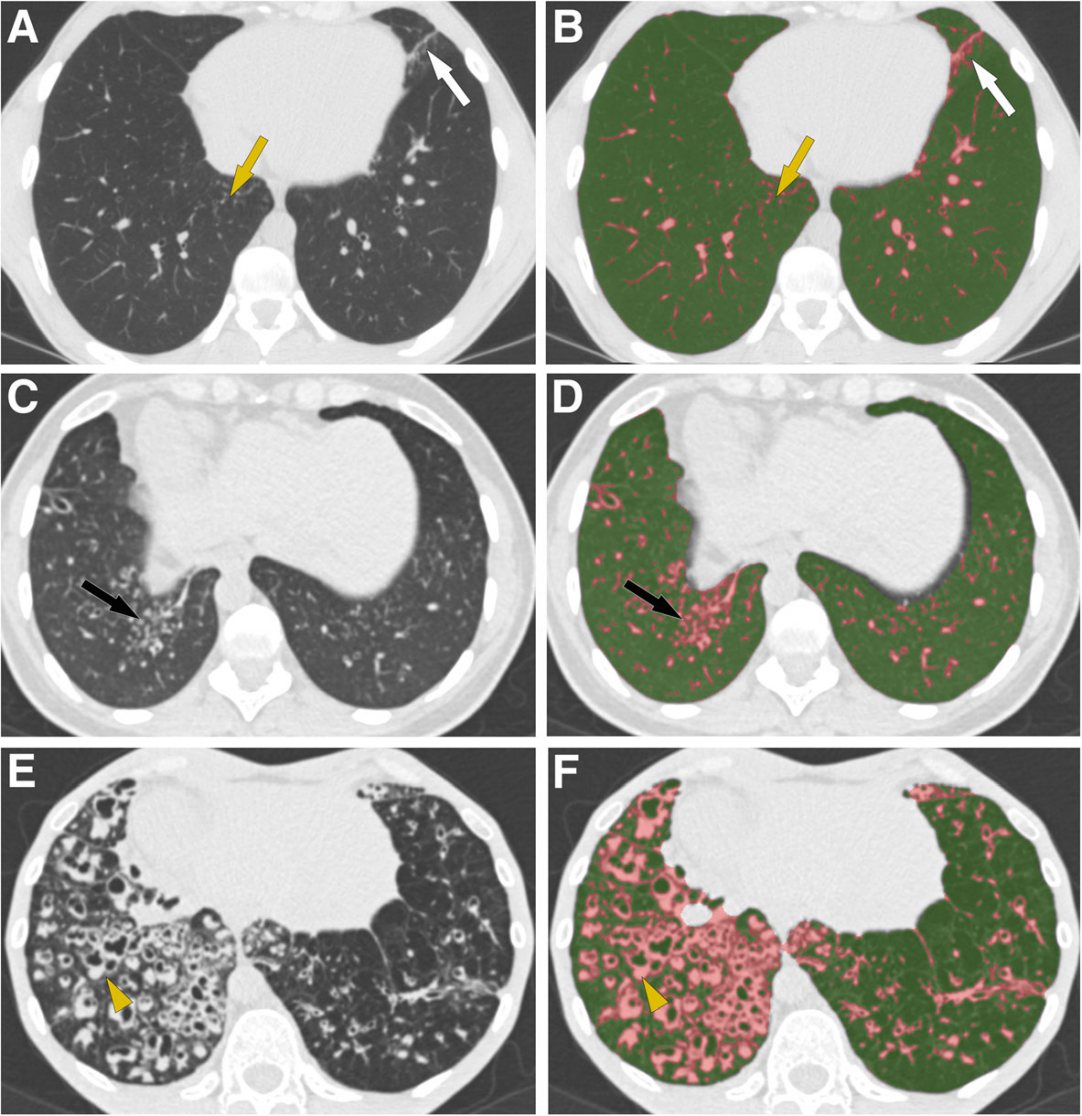
CT-density scores in patients with increasing disease severity. Areas of high attenuation, using MLD + 1SD as threshold, are tagged in pink. **a**, **b** Patient with mild lung involvement (FEV_1_ = 80%, CT score = 6.01): (**a**) Small areas of tree-in-bud (yellow arrow) and subsegmental atelectasis (white arrow) are seen on the native axial image. **b** Post-processed image, illustrating that these PCD-related abnormalities appear in pink, along with pulmonary vessels. **c**, **d** Patient with moderate lung involvement (FEV_1_ = 60%, CT score = 8.37): areas of tree-in-bud (black arrow) seen on the native axial CT image (**c**) are more extensive and are pink-colored on the post-processed image (**d**) (**e**, **f**) Patient with severe lung involvement (FEV_1_ = 25%, CT score = 13.72): (**e**) Visually, bronchial abnormalities (yellow arrowhead) predominantly affect the right lung on the native axial CT image. **f** Post-processed CT image. The CT-density score is 17.59 for the right lung and 10.87 for the left lung

**Fig. 5:**
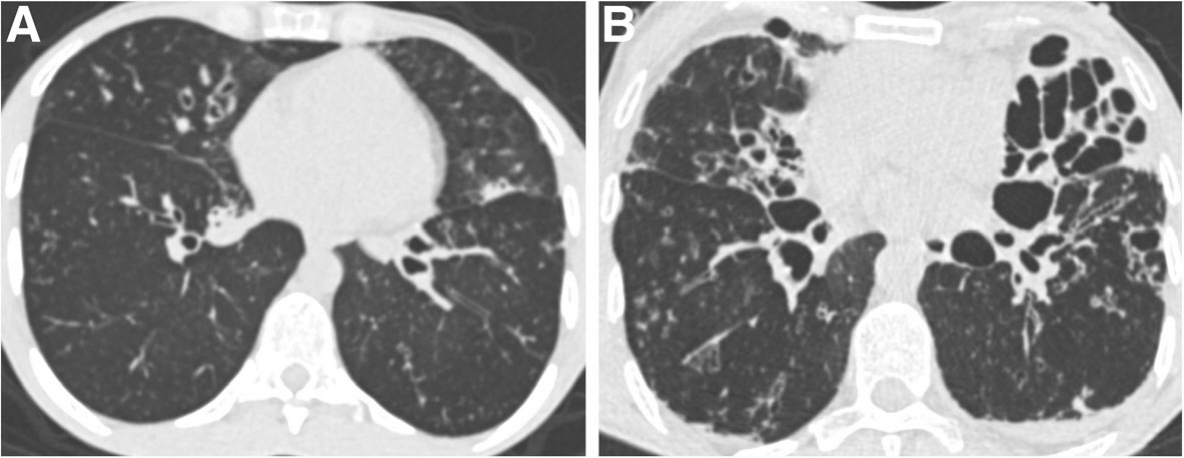
Different imaging features in patients with similar FEV_1_ values. **a** Patient with FEV_1_ = 38% predicted and moderate bronchiectasis predominantly affecting the middle lobe. The CT-density score based on MLD + 1SD was 7.79. **b** Patient with FEV_1_ = 38% predicted but much more severe bronchiectasis on visual assessment, especially in the lingula. The CT-density score based on MLD + 1SD was 12.75. These two examples show that CT imaging provides additional information to spirometry, especially regarding regional disease distribution (homogeneous versus heterogeneous), and the severity of bronchiectasis, which correspond to irreversible changes

Results of automated CT scoring in patients with different FEV_1_ and FVC values are illustrated in Figs. 4 and 5.

## Discussion

To the best of our knowledge, the present manuscript describes the first automated CT scoring method designed to quantify lung changes associated with primary ciliary dyskinesia, based on the measurement of high-attenuating structures and considering histogram characteristics on CT. This approach is close to the quantification of emphysema, based on the measurement of low-attenuating lung areas. The method presented here has been previously validated in CF patients [17].

In the present cohort of 62 adults with PCD, the automated score correlated well with lung function (FEV_1_ and FVC). Moreover, the value of the score was significantly different in the lower and upper portions of the lung, with higher score values in the lower part, consistent with the reported lower lung predominance of bronchial abnormalities in PCD patients [11].

Because the decline of lung function is slower in PCD than in CF, CT is less often performed. However, when performed, lung structural changes on follow-up CT need to be compared to previous images, which is complex in view of the high number of CT images with the modern multidetector technology. Rather than a subjective and time-consuming assessment, automated scoring provides an objective quantitative evaluation.

Regarding the thresholding method, we found that adapted thresholds based on histogram characteristics correlated better than fixed thresholds with the spirometric parameters. Lung attenuation is known to be influenced by parameters such as the level of inspiration, the kilovoltage, and the patient’s position in the scan [21, 25, 26]. Expiration and, by extension, a lower level of inspiration, tend to flatten the CT density histogram, resulting in a higher SD and shifting of the curve towards higher density values, which increases Mode and MLD [26]. Instead of evaluating fixed thresholds alone, we postulated that inclusion of histogram characteristics in the threshold definition would compensate for variations not due to disease severity. Among the various thresholds tested here, MLD + 1SD gave the best results. This threshold is readily available, as most commercially available segmentation software programs provide both MLD and SD values.

One-third of our patients had previously undergone complete or partial lobectomy, even though bronchial abnormalities are not usually restricted to a single lobe in PCD and surgical resection is currently not considered an appropriate treatment for PCD [3]. This proportion is in line with the 41% prevalence reported by Kennedy et al. In this latter study, where visual CT scoring was performed, a maximal score was arbitrarily affected to the missing lung [14]. In our study, we only applied scoring to the existing lung and found good correlations of the visual score to FEV_1_ and FVC, in the upper range of previously reported correlations (0.08 to − 0.63 for FEV_1_ and − 0.38 to − 0.60 for FVC) [8, 10, 12]. The correlation of the CT-density score to FEV_1_ and FVC was in the same range, with the advantage of an automated method for the CT-density score.

Because most structural changes (e.g., bronchial wall thickening, mucus plugging) in PCD increase lung density, we based our automated scoring on the identification of high attenuating structures, leaving out empty bronchiectasis and mosaic perfusion. Thus, the automated score mainly quantifies potentially reversible, inflammatory changes such as mucoid impactions, bronchial wall thickening, bronchiolar nodules and consolidations, whereas it does not consider irreversible changes (e.g., bronchiectasis). Based on these characteristics, we speculate variations in this score may prove useful in identifying worsening of bronchial disease (e.g., during pulmonary exacerbations) or improvement in bronchial patency (e.g., after recovery from a pulmonary exacerbation or due to the beneficial effect of therapy). Due to the unavailability of follow-up chest CT exams for most patients, we were not able to test this hypothesis and future longitudinal studies are needed.

Using our thresholding method, one drawback is that the pulmonary vessels are incorporated into the high-attenuating lung volume, which does not therefore correspond only to the diseased lung. However, differences in pulmonary vessel volume among patients had probably little influence on score variations compared to those due to the bronchial disease, given the good correlation between our score and spirometric parameters. Software capable of pulmonary vessel volume segmentation is being developed [27] and could be used to exclude the pulmonary vessels and improve the performance of the score.

Even though the automated score correlated well with the evaluated functional parameters, we also found that patients with similar FEV_1_ values could have quite different CT phenotypes. We believe that quantitative assessment of structural changes is of interest in addition to PFT measurements for both cross-sectional evaluation and disease monitoring. For example, disease progression in CF has long relied on assessment of lung function decline whereas CT scan analysis clearly shows that structural abnormalities may appear without significant changes in FEV1 [28]. Thus, CT provides structural information which is complementary to spirometry in patients with CF and similar findings are likely occurring in patients with PCD. Calculating CT score does not imply additional procedures for the patients since it can be done from standard CT acquisitions performed as standard of care.

Our study has several limitations. Because this study was retrospective, the CT acquisition parameters were not standardized. This may have influenced the density thresholds. Standardized scanning protocols would probably improve the performance of the developed score. However, the fact that the scoring method can be applied to unstandardized CT examinations makes it suitable for daily clinical use. We did not perform longitudinal evaluation to determine whether changes in the automated CT score correlated with changes in pulmonary function, and whether, as previously suggested, CT-scored disease extent can predict the subsequent decline in pulmonary function. Indeed, our primary objective was to develop and validate an automated CT score by cross-sectional evaluation. Lastly, due to the relative rarity of PCD, it was not possible to split our population into a development and validation cohort. Thus, the developed method should further be validated in an independent cohort of PCD patients.

## Conclusion

In conclusion, automated density–based CT scoring, together with histogram characteristic analysis, is feasible in PCD patients and correlates well with FEV_1_ and FVC. MLD + 1SD offered the best correlations with both FEV_1_ and FVC. Quantitative analysis of structural abnormalities on CT scans may prove useful for objectively evaluating lung disease changes in PCD, which may prove useful both in daily clinical use and as an outcome in clinical trials.

## Abbreviations

CF: Cystic fibrosis
CT: Computed tomography
DLP: Dose-length product
FEV_1_: Forced expiratory volume in 1 s
HU: Hounsfield units
ICC: Intraclass correlation coefficients
MLD: Mean lung density
PCD: Primary ciliary dyskinesia
SD: Standard deviation
